# Association between availability of children’s book and the literacy-numeracy skills of children aged 36 to 59 months: secondary analysis of the UNICEF Multiple-Indicator Cluster Surveys covering 35 countries

**DOI:** 10.7189/jogh.09.010403

**Published:** 2019-06

**Authors:** Alexander Manu, Fernanda Ewerling, Aluisio JD Barros, Cesar G Victora

**Affiliations:** 1 London School of Hygiene & Tropical Medicine, London, UK; 2 Centre for Maternal and Newborn Health, Liverpool School of Tropical Medicine, Liverpool, UK; 3 International Centre for Equity in Health, University of Pelotas, Pelotas, Brazil

## Abstract

**Background:**

Stimulating home environments that have children’s books, pictures and play toys facilitate caregiver-child interactions and enhance children’s development. Although this has been demonstrated in small-scale intervention studies, it is important to document whether book ownership is beneficial at large scale in low and middle-income settings.

**Methods:**

We conducted a secondary analysis using data from the multiple-indicator cluster survey, covering 100 012 children aged 36-59 months, from 35 countries. The outcome was children being on-track for a literacy-numeracy index (LNI) constructed from three questions assessing children’s ability to identify/name at least 10 letters of the alphabet, read at least four simple popular words and know the names and symbols of all numbers from 1-10. The main exposure was availability of children’s book to the child within household. Analysis considered the survey design, assessed and ranked risk ratios of being on track, adjusting for potential confounders such as child’s age (in months), maternal education, household wealth index quintile and area of residence (rural/urban). Ecological analysis was performed using meta-regression after grouping countries by World Bank income groups (low- to high-income).

**Results:**

Only half (51.8%) of children from all the countries analysed have at least one children’s book at home and less than one-third (29.9%; 95% confidence interval (CI) = 23.5%, 36.3%) are on track for literacy-numeracy. After adjusting for confounders, the likelihood of being on track in literacy-numeracy almost doubled if at least one book was available at home compared to when there was none: RR = 1.89 (95% CI = 1.75, 2.03). There was an economic gradient showing that the likelihood of children being on track for LNI decreased with the country’s income group: adjusted-RR ranged from 1.65 in upper middle income to 2.23 in LIC (F-test *P*-value <0.0001). Only three high-income countries were included, and children’s books were universally available resulting in wide confidence intervals for the effect.

**Conclusions:**

These findings are policy-relevant, as they corroborate the results from small scale experiments. Making children’s book available to children is a cheap and feasible intervention that could change home dynamics to improve the future economic fortunes of children especially in the poorest countries.

The marked reduction in overall child deaths between 1990 and 2015, occasioned by strategic paradigm shifts in global health policies towards addressing the fourth Millennium Development Goal (MDG-4), teaches us that when global focus converges and we all commit to a course, substantial progress can be achieved [1,2]. MDG-4 improved visibility of child deaths, stimulated political will to scale-up tested interventions and increased development assistance to high-burden settings [3-5]. In the era of the Sustainable Development Goals (SDGs), child survival is only meaningful if coupled with equitable access to resources for their development to reach optimum potentials. Every year, an estimated 249 million children, mainly from low- and middle-income countries (LMICs), fail to reach their development potential because resources to ensure optimal growth and developmental trajectories are lacking; or when available, scanty and inequitably distributed [6,7]. The dearth of data on children’s cognitive and social-emotional development, as evidence base for interventions for early childhood development (ECD) programming, in most LMICs, further blurs global visibility of the problem [8]. Early childhood investments to develop cognitive and social-emotional skills are known to provide the basis for later economic productivity through higher academic and employment success [9,10].

Effective ECD interventions must reach children very early (within the first three years) when the brain’s neuronal foundations for learning are being laid [6,11]. Early learning, including home opportunities for children to explore and learn and availability of books, toys and play materials, is one of the pillars of the life course conceptual framework for ECD [6,11]. This pillar underscores the role of stimulating home environments and implicitly promotes caregiver involvement in any programme aimed at providing children stable, responsive, and nurturing caregiving; safe, supportive physical environment and appropriate nutrition in these early years [12]. In resource-limited settings, the critical question is: what cheap, effective and feasible intervention(s) can be implemented at homes in early childhood to positively influence children’s developmental trajectories?

Studies in high-income settings show that book-sharing interventions, in which caregivers engage in interactive reading with children, are relatively cheap, feasible to implement and improve social-emotional and cognitive skills [13-17]. Such improvements depend on the quality of the book, their age-appropriateness and how they are used by caregivers to facilitate stimulating interaction with children [18]. Three studies in LMICs [19-21] have also confirmed the benefits of book-sharing interventions on children’s cognitive skills, receptive and expressive language and sustained attention, the latter being a strong predictor of children’s later intelligence quotient (IQ). However, children’s books must first be available at home for such interactions to occur.

When any children’s book is available at home, it is likely to promote caregiver-child interactions that may be stimulating and promote the development of emergent literacy and numeracy skills [18,22]. Evidence suggests that availability of books at home and sharing of books promote increased parental sensitivity and reciprocity and these mediate the linkage with the improved cognitive outcomes for the child [20,23]. Although other factors in the home environment such as parental education may contribute to how the availability of books may affect literacy and numeracy skills, no study to date has described the association between the availability of children’s books at home and children’s literacy and numeracy skills as a composite. It is also plausible that the non-availability of interventions for development may mean that children in LMICs will have larger capacity to develop (or wider ‘zones of proximal development’). Higher improvements in social-emotional and cognitive outcomes have already been found in deprived compared to non-deprived children [10]. Therefore, we hypothesize that the availability of books at home might have a greater beneficial effect in LMICs than in high-income settings [24].

We examined the association between the availability of children’s books within households and literacy and numeracy skills of 36-59-month-old children, using an ecological design. We assessed the trends in this relationship by countries’ wealth status using World Bank national income classifications. If these positive relationships are confirmed at the ecological level, this study will contribute evidence in support of a potentially simple, feasible, scalable and sustainable intervention to improve children’s health, care and development.

## METHODS

### UNICEF Multiple Indicator Cluster Surveys: Data Source

We conducted a secondary analysis using the data from multiple-indicator cluster survey (MICS) from 35 countries for which data was publicly available as at July 2016. The MICS is an international, cross-sectional data collection system implemented by UNICEF to generate internationally comparable data on key indicators mainly focused on maternal and child health. Data collection generally covers the whole country, but can also focus in particular regions. Sub-national surveys were excluded from the analyses. The complex sampling design is standardized across the surveys, guaranteeing the comparability of the results. MICS implemented a module on caregiver-reported early childhood development index (ECDI) since the fourth round of surveys, starting in 2009. The ECDI module collected caregiver-reported data on various exposures and outcomes including availability of children’s books, toys and playthings, supervised care, attendance to early childhood education, support for learning (reading, singing, story-telling), playing with children and assessed the literacy and numeracy skills and behaviours of all children.

### Participants

A total of 100 012 children 36-59 months old children from seven world regions were included in the analysis. There were eight countries from Latin America and the Caribbean (in Argentina, the sample was restricted to urban areas), nine from Central and Eastern Europe & the Commonwealth of Independent States (CEE & CIS), two from South Asia (the 3rd Pakistan Balochistan was sub-national data and was excluded from the analysis), seven from West & Central Africa, four from Eastern & Southern Africa, four from the Middle East & North Africa and two from East Asia & Pacific. Across the countries included in this analysis, differences exist in pre-school opportunities for children as well as the formal educational system. Relevant differences will be discussed in the next sessions.

### Outcome, proximal and distal determinants

**Outcome:** The main outcome for this analysis was children’s Literacy-Numeracy Index (LNI). This was a dichotomous outcome derived from responses to three main questions in MICS that assessed whether children aged 36-59 months: (1) can identify/name at least 10 letters of the alphabet; (2) can read at least four simple popular words and (3) know the names and recognize the symbols of all numbers from 1-10. Considering the operationalization of the ECDI, children were classified as “on-track” for LNI when they passed at least two of the three criteria.

**Main exposure and distal determinants (confounders):** The main exposure or proximal determinant was the availability of children’s or picture books. Four *a priori* confounding variables, were incorporated in the analyses: children’s age (in months), maternal education, household wealth index quintile and area of residence (rural/urban).

1. *Maternal education*: There were variations in the categories for the classification of maternal education across the countries. For instance, in Jamaica, Kyrgyzstan and Moldova, women with no formal education were combined with those who had only basic primary education. In Kyrgyzstan and Moldova, therefore, we compared women with either no formal education or up to the basic primary level education with those who had professional primary/middle, basic/complete secondary and higher education. In Belarus, maternal education was classified into general basic, vocational/technical, general secondary and higher levels. However, in Ukraine, all women who responded to the module were educated up to the secondary level or higher; the categories being secondary/PTU, professional technical, technical, and higher. In contrast, women with no education were combined with those who had only Koranic education in Mauritania where none of the respondents was educated up to the secondary level. These women with no or only Koranic education were compared to those with primary education.
2. *Wealth quintiles*: Wealth index is calculated based on the ownership of household goods and amenities such as (but not being restricted to) persons per sleeping room; type of floor, roof, wall, cooking fuel, and sanitary facility; and source of drinking water using principal components analysis. Based on this, households were then divided into wealth index quintiles. These quintiles are assigned to households in the entire MICS dataset for each country. Households were then selected, based on the availability of children between 36-59 months for the ECDI module. Generally, poorer households have many more children than richer ones and therefore the distribution of children assessed in the ECDI module was not a fifth (20%) across all wealth quintiles as would have been the case if the wealth index was calculated only with the children included in the ECDI module.
3. *Age and sex of the child*: We included the child’s age, in months, as a potential confounder given that the outcome most likely will increase with the age of the child. We also explored whether sex was a confounder or an effect modifier.
4. *Place of residence*: Within a country, residing in a rural or urban area affects access to various services including exposure to stimulating environments at home.
5. *Stunting*: We estimated stunting as children whose height-for-age was more than 2 standard deviations from the standardized mean using the WHO standards.

### Statistical analysis

We conducted the analyses using the statistical software Stata® (Stata Corp, Texas, USA version 14.0). Simple and cross tabulations were performed, taking the study design into consideration in all analyses by using Stata’s “svy” command prefix to account for sample weights, clustering, and stratification.

All countries had data on the number of books available to the child, except Mauritania where the information was dichotomous (“yes or no”). We therefore excluded Mauritania, categorised the variable into: no book, only 1 book and 2 books or more and assessed trends in the likelihood of being on track for LNI by number of books. However, the median number of books was zero in 56% of the countries. An additional 5% of countries had a median of 1 children’s book. We therefore dichotomised availability of children’s books into those with at least one book compared to those with none, which allowed for inclusion of Mauritania in the analyses.

At the country level, we estimated the proportion of children on track for LNI as well as those with at least one children’s book available and ranked countries by these proportions. We then fitted for each country a Poisson regression with robust variance to estimate crude risk ratios (RR) and 95% confidence intervals (95% CI) of the children being on track for LNI according to the availability of books. The models were adjusted for the distal determinants (children’s age in months, wealth index quintiles, area of residence and maternal education) to generate adjusted RRs (and their 95% CIs) for each country. We explored role of stunting in the relationship between book ownership and LNI and found that stunting was not a confounder.

For the ecological analysis, we grouped the countries according to The World Bank 2016 fiscal year income classification into Low- (LIC, 8 countries), Lower Middle- (l-MIC, 11 countries), Upper Middle- (u-MIC, 13 countries) and High-income (HIC, 3 countries) groups. We tabulated the data for each country to obtain the percentage of households that responded to the ECDI module within each category of the distal determinants. Medians and interquartile ranges of these percentages were used to represent the distribution of these determinants within the World Bank income groups.

Aggregate effect estimates were generated for the association between LNI and availability of children’s books. To do this, we considered that, since policy and social contexts of each of the constituent countries differed within and between World Bank income groups, data from each individual country represents a ‘study on their own’. We therefore fitted random effects meta-regression models to aggregate the effects by these groups. The random effects models allowed for within and between group variations and was most appropriate to test whether the effect of having children’s book available to a child on their likelihood of being on track for LNI depended on the country’s income group and whether the effect sizes were homogeneous within and between groups. The main effect of World Bank income group was also fitted in the model and likelihood ratio tests were performed to assess statistical significance. However income group was not fitted as interaction term in the model . Funnel plots were done to assess for any evidence of heterogeneity in the pooled effect estimates within each of the groups and overall.

### Ethical considerations

MICS are publicly available, so permission for access and use of these data are not required. Ethical clearance was obtained by UNICEF and their implementing partners within countries by the time of the surveys’ conduction.

### Role of the funding source

The Countdown to 2030 Equity Technical Working Group sponsored this analysis with funding from the Wellcome Trust [Grant Number: 101815/Z/13/Z]; Bill & Melinda Gates Foundation [Grant Number: OPP1135522]; and Associaçăo Brasileira de Saúde Coletiva (ABRASCO).

## RESULTS

The 35 countries that implemented the ECD module in their MICS are shown in **Table 1**. In total, 100 012 children (36-59 months old) were assessed for the literacy and numeracy index (LNI) in these MICS surveys. Across all the countries, the mean proportion of children on track for LNI was 29.9% (95% CI = 23.5%, 36.3%). **Table 1** shows ranking of countries based on percentage of children on track for LNI. There was a very strong positive correlation between World Bank income group and LNI (Pearson r = 0.66; 95%CI = 0.42, 0.81). Only the top three highest ranked countries had up to half (50%) of children on-track for the LNI. In the four countries ranked lowest for LNI, approximately nine out of every ten children were not on track for LNI. It is important to note that the seven lowest ranked countries were from sub-Saharan Africa and had less than 15% of children on track for LNI.

**Table 1:** The 35 countries, UNICEF-MICS region & World Bank income group ranked by proportion of children on track for Literacy-Numeracy Index (LNI)

Country	UNICEF MICS Region	2016 World Bank income group	Sample size	Children on track for LNI		Children with ≥1 book		Trend in LNI by No. of books-3 categories, *P*-value	RR (95% CI) of LNI by availability of books	
**Weighted %**	**Rank**	**Weighted %**	**Rank**	**Crude**	Adjusted*
Chad	West & Central Africa	Low	7022	5.7	35	2.9	35	<0.0001	6.59 (4.71, 9.21)	3.19 (2.20, 4.62)
Central African Republic	West & Central Africa	Low	3726	7.1	34	4.9	33	<0.0001	5.81 (4.05, 8.32)	2.26 (1.47, 3.47)
Sierra Leone	West & Central Africa	Low	3675	9.4	33	10.1	31	<0.0001	4.90 (3.84, 6.25)	2.40 (1.79, 3.22)
Zimbabwe	East & Southern Africa	Low	1070	9.6	32	14.8	29	<0.0001	4.22 (3.50, 5.08)	2.78 (2.25, 3.43)
Togo	West & Central Africa	Low	1792	10.7	31	10.3	30	<0.0001	4.28 (3.10, 5.91)	2.05 (1.40, 2.99)
Democratic Republic of Congo	East & Southern Africa	Low	4039	10.8	30	3.9	34	<0.0001	2.86 (2.02, 4.05)	1.94 (1.39, 2.71)
Swaziland	East & Southern Africa	Lower Middle	1070	14.8	29	15.4	28	<0.0001	4.31 (3.15, 5.88)	2.71 (1.90, 3.85)
Kyrgyzstan	Eastern Europe	Lower Middle	1780	14.9	28	63.8	16	<0.0001	3.29 (2.30, 4.69)	2.06 (1.45, 2.93)
Malawi	East & Southern Africa	Low	7687	17.6	27	4.9	32	<0.0001	3.41 (2.89, 4.02)	1.99 (1.67, 2.37)
Iraq	Middle East & North Africa	Upper Middle	13894	17.9	26	16.4	27	<0.0001	3.08 (2.69, 3.53)	2.06 (1.74, 2.43)
Mauritania	West & Central Africa	Lower Middle	3679	19.3	25	35.9	21	Not applicable†	3.00 (2.56, 3.52)	1.76 (1.49, 2.07)
Lao PDR	East Asia & Pacific	Lower Middle	4457	19.8	24	19.6	26	<0.0001	4.03 (3.49, 4.65)	1.87 (1.59, 2.19)
Suriname	Latin America & Caribean	Upper Middle	1276	20.8	23	45.5	20	<0.0001	2.39 (1.79, 3.19)	1.47 (1.07, 2.02)
State of Palestine	Middle East & North Africa	Upper Middle	3219	22.5	22	48.3	19	<0.0001	2.53 (2.16, 2.95)	1.99 (1.71, 2.33)
Montenegro	Eastern Europe	Upper Middle	645	23.7	21	92.5	5	<0.0001	8.28 (2.03, 33.79)	4.69 (1.09, 20.2)
Bhutan	South Asia	Lower Middle	2420	24.6	20	20.6	25	<0.0001	3.23 (2.71, 3.83)	1.97 (1.58, 2.44)
Bosnia	Eastern Europe	Upper Middle	1030	25.1	19	87.5	10	<0.0001	1.70 (0.78, 3.70)	1.36 (0.67, 2.74)
Costa Rica	Latin America & Caribean	Upper Middle	903	27.3	18	73.0	14	<0.0001	2.94 (1.72, 5.02)	2.09 (1.17, 3.74)
Ghana	West & Central Africa	Lower Middle	3067	28.3	17	24.2	24	<0.0001	3.02 (2.54, 3.59)	1.52 (1.22, 1.89)
Nepal	South Asia	Low	2249	29.1	16	31.7	22	<0.0001	3.23 (2.72, 3.84)	1.84 (1.54, 2.19)
Kazakhstan	Eastern Europe	Upper Middle	1961	29.5	15	82.9	12	<0.0001	3.21 (2.27, 4.53)	2.38 (1.70, 3.32)
Vietnam	East Asia & Pacific	Lower Middle	1185	29.9	14	48.9	18	<0.0001	2.10 (1.69, 2.60)	1.72 (1.37, 2.15)
Moldova	Eastern Europe	Lower Middle	732	30.4	13	88.0	9	<0.0001	2.71 (1.33, 5.57)	1.78 (0.87, 3.64)
Tunisia	Middle East & North Africa	Upper Middle	1161	31.6	12	52.1	17	<0.0001	2.02 (1.55, 2.65)	1.33 (0.97, 1.83)
Nigeria	West & Central Africa	Lower Middle	10170	32.6	11	26.1	23	<0.0001	4.19 (3.78, 4.64)	1.85 (1.64, 2.09)
Serbia	Eastern Europe	Upper Middle	1190	35.9	10	94.2	4	<0.0001	4.25 (2.06, 8.77)	2.35 (1.00, 5.53)
Argentina	Latin America & Caribbean	High	3602	40.6	9	84.9	11	<0.0001	2.08 (1.65, 2.64)	1.85 (1.45, 2.35)‡
Macedonia	Eastern Europe	Upper Middle	557	43.5	8	76.1	13	0.004	1.35 (0.93, 1.98)	1.24 (0.82, 1.89)
Ukraine	Eastern Europe	Lower Middle	1900	45.8	7	99.5	2	0.589	1.32 (0.47, 3.75)	1.13 (0.46, 2.77)
Belize	Latin America & Caribbean	Lower Middle	788	45.9	6	66.0	15	<0.0001	1.92 (1.51, 2.45)	1.77 (1.38, 2.27)
Belarus	Eastern Europe	Upper Middle	1411	46.9	5	99.8	1	0.691	1.35 (0.51, 3.54)	0.37 (0.14, 0.96)
Uruguay	Latin America & Caribbean	High	744	49.1	4	91.0	6	<0.0001	3.56 (1.90, 6.67)	3.33 (1.60, 6.90)
Jamaica	Latin America & Caribbean	Upper Middle	668	65.8	3	88.5	8	<0.0001	1.67 (1.28, 2.19)	1.40 (1.09, 1.80)
St Lucia	Latin America & Caribbean	Upper Middle	122	70.3	2	88.7	7	0.036	1.70 (1.04, 2.78)	1.25 (0.75, 2.09)
Barbados	Latin America & Caribbean	High	202	89.9	1	99.0	3	0.222	1.41 (0.58, 3.42)	1.40 (0.62, 3.15)§

RR – risk ratio, CI – confidence interval

*The model was adjusted for wealth index quintile, maternal education, rural or urban residence and age of the child (in months).

†Mauritania had a dichotomous outcome for book availability that only assessed whether any children’s book was available or not.

‡The adjusted model excluded rural/urban residence since they were all urban.

§Maternal education not in model.

**Table 2** shows the distribution of the distal determinants by World Bank income group. Overall, 63% of the respondents reside in rural areas but there were differences by income groups; whilst only 8% in HICs reside in rural areas, about three-quarters (74.4%) in LICs reside in rural areas. Also, overall, 23.3% of respondents were in the poorest wealth quintile but there seemed to be a higher representation (28%) of the poorest quintile in HICs compared to the richest (15%). Only 6.8% of mothers had no formal education but again this varies from zero in HICs to just under one-half (46%) in LICs.

**Table 2:** Summary distribution of determinants by World Bank Income groups

World Bank Income Group	Number of countries	Percentages of distal determinants: median (interquartile range, IQR)			
**Wealth Index Quintile (WIQ)**		**Rural residence**	**Mothers with no formal education**
**Poorest WIQ**	**Richest WIQ**		
High Income (HIC)	3	27.8 (20.2, 37.6)	14.8 (13.1, 18.9)	7.7 (0.0, 37.1)	0.0 (0.0, 0.4)
Upper Middle Income (l-MIC)	13	22.4 (17.9, 24.3)	17.1 (14.8, 23.1)	39.7 (34.6, 49.4)	1.7 (0.6, 4.1)
Lower Middle Income (u-MIC)	11	23.6 (21.7, 25.0)	16.6 (14.5, 18.8)	70.6 (62.1, 72.8)	18.8 (4.9, 46.7)
Low Income (LIC)	8	22.7 (20.8, 23.6)	16.0 (14.9, 16.6)	74.4 (70.5, 83.7)	45.8 (21.2, 62.6)
**Overall**	**35**	**23.3 (20.3, 24.3)**	**16.4 (14.5, 18.8)**	**62.5 (36.6, 73.4)**	**6.8 (0.9, 36.6)**

On average, 51.8% (95% CI = 39.8%, 63.7%) of respondents had one or more children’s books available to the 36-59-month-old child (**Table 1**). The percentage with at least one children’s book available to the child seemed to increase with the wealth of the country based on World Bank income grouping. A median (IQR) of only 7.5% (4.4%-12.6%) of households in LICs assessed for ECDI had at least one children’s book available to the child as compared to 35.9% (20.6%-66.0%) in l-MICs, 82.9% (52.1%-88.7%) in u-MICs and 91% (84.9%-99.0%) in HICs. **Table 1** also shows the ranking of countries by the percentage of households with at least one children’s book to the child. In the 6 top-ranked countries (Belarus, Ukraine, Barbados, Serbia, Montenegro and Uruguay), over 90% of the households had at least one (usually more) children’s book available to the child. The two lowest ranked countries (Chad and Central African Republic) were exactly the same as those with the lowest rank for LNI.

**Figure 1:**
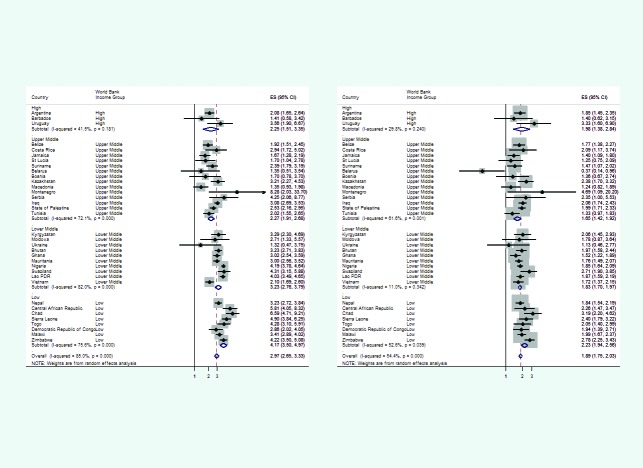
Funnels plot showing crude (left) and adjusted (right) risk ratios of the association between availability of children's book and being on track for Literacy-Numeracy Index (LNI) by World Bank Income Classification.

There was a strong correlation between the proportion of children on track for LNI and proportion with at least one children’s book available to the child (Pearson r = 0.74; *P* < 0.001; see **Figure 1**). The trend in the ranking of countries based on percentage of households with at least one children’s book to the child was consistent with that for percentage of children on track for LNI. Seven out of the ten lowest ranked countries for both percentage of children on track for LNI and having at least one children’s book available to the child were from LICs. In Chad, CAR, Malawi and Democratic Republic of Congo, less than 5% of households had children’s books available to the child. In contrast, apart from Belize and Ukraine (l-MIC), all 10 top-ranked countries with respect to percentage on track for LNI were HICs or u-MICs. Similarly, apart from Belize, the top 8 countries in terms of percentage with children’s book available to the child were also from HICs or u-MICs.

The pooled unadjusted likelihood of children being on track for LNI when at least one children’s book is available to the child increases approximately 3-folds compared to when there is none (unadjusted-RR = 2.97; 95% CI = 2.65, 3.33; *P* < 0.001). **Figure 1** shows that the unadjusted-RR varies from a non-statistically significant 32% increase in Ukraine to more than 8-fold increase in Montenegro although the latter had very wide confidence intervals consistent with a possible 34-fold increase in the likelihood. There was a clear trend in the likelihood by country’s income class; unadjusted-RRs increase from 2.25 in HICs to 4.17 in LICs. **Table 3** shows that the overall variability in the outcomes attributable to heterogeneity between countries was 85.0% (with *P* = <0.0001). The trends, however, were in one direction across all countries-pointing towards higher likelihood of being on track for LNI when any children’s book was available to the child.

**Table 3:** Pooled unadjusted and adjusted RRs for children on track for literacy-numeracy index (LNI) by availability of children’s books to child by World Bank income group for the 2016 fiscal year

World Bank Income Group	Number of countries	Unadjusted risk ratios (95% CI) of children on track for LNI by availability of at least 1 book to child		Adjusted risk ratios (95% CI) of children on track for LNI by availability of at least 1 book to child	
**RR (95% CI)**	I^2*^ (*P* -value)	**RR† (95% CI)**	**I^2^ (*P*-value)**
High Income (HIC)	3	2.25 (1.51, 3.35)	41.5% (0.181)	1.98 (1.38, 2.84)	29.8% (0.189)
Upper Middle Income (l-MIC)	13	2.27 (1.92, 2.68)	72.1% (<0.0001)	1.65 (1.42, 1.92)	61.6% (0.014)
Lower Middle Income (u-MIC)	11	3.24 (2.76, 3.79)	82.0% (<0.0001)	1.83 (1.70, 1.97)	11.0% (0.001)
Low Income (LIC)	8	4.17 (3.50, 4.97)	75.6% (<0.0001)	2.23 (1.94, 2.56)	52.6% (0.031)
**Total**	**35**	**2.97 (2.65, 3.33)**	**85.0% (<0.0001)**	**1.89 (1.75, 2.03)**	**54.4% (<0.0001)**

RR – risk ratio, CI – confidence interval

*I^2^ is the percentage of the total variation that is due to heterogeneity between the countries’ RR.

**†**RR adjusted for wealth index quintile, maternal education, rural or urban residence and age of child (in months).

When adjusted for household wealth index quintile, place of residence, maternal educational attainment, and the child’s age (in months), the pooled adjusted likelihood of being on track for LNI when any book was available to the child was almost 2-fold compared to when there was no book: adjusted-RR = 1.89 (95% CI = 1.75, 2.03). The variability attributable to differences between countries, I^2^, is now 54%, being mainly driven by the high variability in u-MICs (I^2^>60%). **Table 1** and **Figure 1** show that Montenegro and Uruguay had an unexpectedly high adjusted-RRs (4.69 and 3.33, respectively) because very few households had no children’s books available to children, leading to very wide confidence intervals around the estimate. Apart from the latter, the top four countries with the highest adjusted-RRs were Chad (RR = 3.2; 95% CI = 2.2, 4.6), Zimbabwe (RR = 2.8; 95% CI = 2.3, 3.4), Swaziland (RR = 2.7; 95% CI = 1.9, 3.9) and Sierra Leone (RR = 2.4; 95% CI = 1.8, 3.2), all LICs or l-MICs. **Table 3** also demonstrates that, irrespective of the wealth index quintile, maternal education, area of residence and children’s age, the adjusted-RR also seems to decrease with the country’s income group: adjusted-RR increases from 1.65 in u-MIC to 2.23 in LIC. The country’s income group was tested in the model exploring the relationship between being on track for LNI and having children’s book available to the child and showed that, at the ecological level, this was statistically significant (F-test *P*-value <0.0001).

## DISCUSSION

To our knowledge, this study is the first to systematically quantify the association between having at least one children’s book for 36-59 months old children at home and their literacy and numeracy skills development in LMICs. Our results show that, irrespective of maternal education, wealth index quintile, children’s age (in months), and area of residence, having at least one children’s book to a child almost doubles their likelihood of being on track for literacy-numeracy: adjusted-RR = 1.89 (95% CI = 1.75, 2.03). There was also a suggestion of an economic gradient: the adjusted-RRs were highest in LICs (RR = 2.23) and reduced with increasing countries’ income (LR-test of the main effect of WBIC: LR χ2(df = 3) = 108.3; *P* < 0.0001). This may be an indication that the potential of improving children’s literacy and numeracy skills by making children’s books available is greatest in LICs.

Our analysis is limited by the number of available relevant confounders that were collected in the MICS surveys. The reduction in the RR from 2.97 to 1.89 after adjusting for the confounders may suggest that there could still be some residual confounding that might not have been adjusted for and therefore interpretation of the results requires caution. In particular, we do not have information on whether there are older children in the household to whom the books may belong. Stunting was not found to be a confounder. We thought that stunting could therefore be a mediator of wealth or other nutritional determinants early in life but this analysis is beyond the scope of this analysis.

Our results do not imply causality or that making children’s books available to households is the only requirement for improving children’s literacy and numeracy skills and their consequent cognitive development. It however builds on the evidence from small-scale studies (mainly from HICs and a few from LMICs) that demonstrated improvements in children’s linguistic, socio-emotional and cognitive development when books are distributed to households or interactive/dialogic reading with children is promoted [13-17,19-21,25]. It is not uncommon for interventions that work well at small scale to be found ineffective when scaled up [26]. It is therefore reassuring that the results of existing small studies are replicated in large populations, so these results cannot be overlooked.

This consistency with global trends in child survival, growth and development is significant and, though the results only demonstrate a cross-sectional association, they might have important programmatic implications. These observed trends between availability of any children’s book to the child and the foundations of their literacy-numeracy development across country’s income levels have possible theoretical basis. Vygotsky’s will see children in the poorest settings as having a large, untapped ‘zones of proximal development’ [24]. When books are available to the child, they provide platforms to ‘scaffold’ opportunities for stimulating interactions with children and consequently their literacy and numeracy skills development. Linguistic development is one of the four key domains identified by the World Bank’s Systems Approach for Better Education Results (SABER) framework for intervention in the early years in order to impact on life outcomes [27].

Increasingly, developing countries are formulating new policies on child development with the help of global partners like the World Bank [28]. Ensuring that every child in every household has access to at least one children’s book is potentially feasible, easy, and cheap to implement. Identifying existing platforms to implement such relatively simple (both within communities and at facilities) such as postnatal visits to static facilities or mobile outreach clinics, home visits by community-based agents or women’s groups and supporting these with effective community mobilization [28-30] may be effective, equitable and sustainable but needs to be tested.

Our analysis has several strengths; the data sets are nationally representative MICS, which has been one of the major sources of data used by UN agencies to generate sensitive maternal and newborn health indicators in low-resourced settings. The MICS design and questionnaires were highly standardized, and countries from all economic groups were represented, even though the numbers were relatively small for HICs resulting in lower statistical power [31].

There have been some questions raised about the robustness of the UNICEF Early Child Development Index in measuring the status of child development [32]. However, this index that measures the developmental status of children within four domains: literacy-numeracy, physical, and social-emotional development is one of the only global sources to measure children’s outcomes in countries of all income classes in the early childhood years [33]. The index and its components were developed using robust methods that involved validation with widely-used tools like the Strengths and Difficulties Questionnaire [34] and the Early Development Instrument [35,36] followed by factor analyses, expert consultation and filed testing of the final 10-item tools that included these three questions on literacy and numeracy assessment. Whilst UNICEF acknowledges that these measures are work in progress and have been working with the experts at the WHO, academics and programme implementers to make the measures more robust, they still provide a compelling case for more effective, and targeted interventions as well as for advocacy to prioritise early child development in global public health investments [33].

Educational systems also differ across countries and therefore the maternal educational levels in the MICS data set may represent different levels of literacy in different countries. Moreover, in most high and upper middle-income countries, pre-school educational systems are better established than in LMICs [27,37] increasing the likelihood that children will have books at home. Asset-based wealth indices have been criticised because of challenges in comparing assets between rural and urban settings, but the formulations of the indices used in the present analyses takes such differences into account [38], and asset-based wealth indices have been found to perform very well in many settings [39]. Whilst context and unmeasured confounding cannot be ignored, the observed trends are still quite compelling.

More importantly, the present results mirror existing trends in child survival and undernutrition between regions and across national income classes and hence may be legitimate. Countries with the highest RRs such as Chad (3.19), Zimbabwe (2.78), Swaziland (2.71) and Sierra Leone (2.40) also present poor overall child health indicators. When at least one book is available to the child, our results show that the potential for improving the likelihood of them being on track for LNI is highest in LMICs, mainly countries from sub-Saharan Africa and South Asia. These concur with findings from other studies that identified those two regions as having the worst ECD indices globally [6,32,40]. Evidence on the effect of book availability on children’s educational attainment also suggest that when families have few or no books at all, additional books produce higher gains in educational attainment than when they already have many [41] and may indicate that interventions to make books available to children will have maximal effect in these settings.

Scaling-up of such interventions will require integrated delivery at optimal quality with universal coverage. If funding is not sufficient for universal coverage, pro-poor targeting will be crucial to avoid the tendency of making only the well-off have access in accordance with the inverse equity hypothesis [42]. In LMICs, particularly, delivery will require a strong political will to effect policy changes, system strengthening, adequate stakeholder engagement (including private sector providers and families), adequate funding and in-built robust accountability mechanisms to monitor quality and progress [43,44]. There is also the need for contextualising such an intervention to promote family involvement and ownership which will then facilitate the next steps beyond availability of books but stimulating parental interactions including reading these books with the children.
